# Depressive symptoms and visual attention to others’ eyes in healthy individuals

**DOI:** 10.1186/s12888-024-05633-2

**Published:** 2024-03-06

**Authors:** Thomas Suslow, Dennis Hoepfel, Anette Kersting, Charlott Maria Bodenschatz

**Affiliations:** Department of Psychosomatic Medicine and Psychotherapy, University of Leipzig Medical Center, Semmelweisstr. 10, 04103 Leipzig, Germany

**Keywords:** Depressive symptoms, Visual attention, Eyes, Facial expression, Face mask, Gaze behavior, Eye-tracking

## Abstract

**Background:**

Eye contact is a fundamental part of social interaction. In clinical studies, it has been observed that patients suffering from depression make less eye contact during interviews than healthy individuals, which could be a factor contributing to their social functioning impairments. Similarly, results from mood induction studies with healthy persons indicate that attention to the eyes diminishes as a function of sad mood. The present screen-based eye-tracking study examined whether depressive symptoms in healthy individuals are associated with reduced visual attention to other persons’ direct gaze during free viewing.

**Methods:**

Gaze behavior of 44 individuals with depressive symptoms and 49 individuals with no depressive symptoms was analyzed in a free viewing task. Grouping was based on the Beck Depression Inventory using the cut-off proposed by Hautzinger et al. (2006). Participants saw pairs of faces with direct gaze showing emotional or neutral expressions. One-half of the face pairs was shown without face masks, whereas the other half was presented with face masks. Participants’ dwell times and first fixation durations were analyzed.

**Results:**

In case of unmasked facial expressions, participants with depressive symptoms looked shorter at the eyes compared to individuals without symptoms across all expression conditions. No group difference in first fixation duration on the eyes of masked and unmasked faces was observed. Individuals with depressive symptoms dwelled longer on the mouth region of unmasked faces. For masked faces, no significant group differences in dwell time on the eyes were found. Moreover, when specifically examining dwell time on the eyes of faces with an emotional expression there were also no significant differences between groups. Overall, participants gazed significantly longer at the eyes in masked compared to unmasked faces.

**Conclusions:**

For faces without mask, our results suggest that depressiveness in healthy individuals goes along with less visual attention to other persons’ eyes but not with less visual attention to others’ faces. When factors come into play that generally amplify the attention directed to the eyes such as face masks or emotions then no relationship between depressiveness and visual attention to the eyes can be established.

**Supplementary Information:**

The online version contains supplementary material available at 10.1186/s12888-024-05633-2.

## Background

Eye contact has been defined as two people gazing at each other’s eyes [1]. Making and modulating eye contact is a fundamental part of human communication at all ages [2]. Healthy people mainly examine the eyes when looking at facial expressions [3, 4]. Eye contact represents a crucial signal in the initiation of conversations [5]. Mutual gaze is assumed to constitute a key factor in facilitating bonding between child and caregiver [6]. Neonates prefer direct to averted gaze [7] and detect eye-like stimuli in the environment orienting their attention toward them [8]. During face-to-face interactions gaze has important social functions: it allows individuals to modulate transitions between speaker and listener states, to track attentional and emotional states of the partner, and to regulate the level of arousal in the interaction [9]. The social meaning of eye contact and its duration is strongly context dependent [10]. Direct gaze can be used to signal social interest and closeness [11, 12] or can be a sign of love and attraction [13], but prolonged gaze or staring can cause avoidance behaviors [14] and be interpreted as expression of dominance depending on the status of the sender [15]. It has been shown that perceived eye contact modulates subsequent cognitive processing [16, 17]: direct gaze captures the beholder’s attention and then enhances self-awareness triggering self-referential processing, i.e., memory for self-relevant information.

Depression is an affective disorder characterized mainly by low mood and loss of positive affect [18]. Social factors are assumed to be involved in the pathogenesis and the consequences of depression [19]. Individuals suffering from depression manifest decreased pleasure and interest in social encounters and tend to withdraw socially [20, 21]. A factor that could contribute to social functioning impairments in depression is reduced eye contact during social interactions. It has been observed that depressed patients make less eye contact during clinical interviews compared to healthy individuals and patients suffering from somatic disorders [22, 23]. Moreover, longitudinal studies based on interviews and hospital ward observations showed that eye contact increases with the improvement of the depressive state [24, 25]. Treatment with antidepressant medication may enhance eye contact during dyadic interactions in depressed patients [26].

There is evidence that eye contact might not only be affected by clinical depression but also as a function of sad mood in healthy individuals. Natale [27] observed that, subsequent to a mood induction procedure, sad individuals had less total eye contact during a conversation than individuals in a neutral affective state. In this study, subject’s returned eye contact was assessed. In contrast, elated individuals engaged in more total eye contact than those in a neutral mood [27]. Hills and Lewis [28] conducted an experiment in which participants made discriminations between faces that had either featural or configural changes made to the eyes, head shape, or nose. Participants induced to be happy detected changes in the eyes more often than those induced to be sad. Sad-induced participants detected changes to the head shape but not the eyes. The authors interpreted their findings in terms of differential use of facial features attended to by sad and happy participants, whereby sad persons are less likely to attend to eyes during face perception than happy persons [28].

There are two eye-tracking studies that investigated the effect of depressive symptoms on gaze behavior during emotion recognition in samples of university students [29, 30]. Based on scores of the Beck Depression Inventory, Hunter et al. [29] formed two study groups by median-split. They asked participants to label facial expressions and measured the visit duration on upper (forehead and eyebrows), middle (eyes and cheekbones), and lower (nose and mouth) facial areas of interest. In this study, recognition performance was not analyzed. Individuals with non-clinical depressive symptomatology were found to view at emotional expressions very similarly to individuals with no depressive symptoms. However, individuals with depressive symptomatology focused more on the lower than the middle area of interest during the perception of fearful, neutral, angry, and sad expressions. It was concluded that these individuals might draw their gaze to lower facial areas of unpleasant emotions to avoid emotional displays, which may match the depressive symptoms. Wu et al. [30] used the Self-Rating Depression Scale [31] to form two study groups and registered participants’ eye-movements during an emotion recognition task. Interestingly, the group with depressive symptoms responded quicker while performing as accurately as the group without depressive symptoms in the emotion recognition task. According to the eye-tracking data the group with depressive symptoms dwelled less time on eyebrows, eyes, nose, and mouth than the group without depressive symptoms. In sum, the results of the eye-tracking studies of Hunter et al. [29] and Wu et al. [30] suggest that depressive symptoms in non-clinical samples could be associated with less attention to facial features, in particular middle facial areas when labeling facial expressions. Both studies did not address the issue of depressive symptomatology and avoidance of direct gaze or eye contact. Free viewing tasks seem to be more adequate to assess natural or spontaneous viewing patterns when people look at faces than goal-directed tasks such as emotion recognition. Free viewing imposes no external constraints on what locations or parts of a stimulus should be looked at. Instead, what locations are interesting or relevant are defined primarily by the observer. Free viewing tasks have been successfully administered to reveal attentional preferences and biases in anxiety and affective disorders [32, 33].

The present eye-tracking study examined whether depressive symptoms in healthy individuals are associated with reduced visual attention to other persons’ direct gaze. To this aim, we compared the gaze behavior between two groups, individuals reporting a series of current depressive symptoms and those reporting no (or hardly any) depressive symptoms. Our investigation could contribute to enhance the understanding of depression-related impairments in social perception. In our study, a free viewing task was administered in which pairs of faces showing emotional or neutral expressions were presented with a direct gaze. This screen-based task allows to assess eye contact related gaze behavior, but it does not examine eye contact in a setting of real face-to-face interaction. One-half of the face pairs was shown without face masks, whereas the other half wore face masks (covering the mouth and parts of the nose). During the COVID-19 pandemic, face masks have been widely used in daily life [34]. It was hypothesized that individuals with depressive symptoms would look shorter at other persons’ eyes in unmasked as well as masked faces. Moreover, it was assumed that dwell time on the eyes of faces with a mask would in general be longer than on the eyes of unmasked faces since there is evidence that people move their gaze preferentially to the eyes of faces in which the lower part is covered [35]. The presentation of masked faces provides an opportunity to explore if an effect of depressive symptoms on fixation of the eyes can still be identified when participants’ gaze is strongly directed towards the eye region. It is conceivable that individuals with depressive symptoms could manifest less attention to the eyes and exhibit avoidance tendencies when they are pushed to look at the eye region by facial stimulus properties. However, it is also possible that depressive symptoms may not exert an effect on gaze orientation in case of faces wearing face masks since there is a strong stimulus-driven effect that attracts and holds attention on the eye region. In our analyses, the mouth was included as a second area of interest to investigate whether depressive symptoms are related to a specific or global reduction of gaze to others’ faces. To examine the above-mentioned research questions on visual attention to the eyes, we could have used an experimental design with the presentation of a single face. However, we were also interested in the question whether the eyes of emotional faces attract more attention than the eyes of simultaneously presented neutral faces. Against this background, we decided to present a combination of two facial expressions with direct gaze, an emotional and a neutral one, in our free viewing task. Finally, we tested whether depressive symptoms are linked to reduced visual attention to the eyes as a function of emotional facial expression (happiness, sadness, fear, and disgust).

## Methods

### Participants

Our final sample consisted of 93 young healthy individuals (64 women). Three participants had to be excluded from data analysis, two due to calibration difficulties and one due to acoustic disturbances during the eye-tracking experiment. Participants were recruited via online social media and public notices, which were posted in libraries, student halls of residence, canteens, and supermarkets. Study participants were native German speakers or spoke German since the age of six. All had normal vision as assessed by a Snellen eye chart test. The majority of study participants were university students (*n* = 67) from diverse academic disciplines. The other participants were working persons (*n* = 15), in vocational training (*n* = 7), in a voluntary social year (*n* = 2), school student (*n* = 1) or unemployed (*n* = 1).

At the beginning of the study, all interested people were given a brief description of the investigation and its procedures. Then, trained doctoral students interviewed them by telephone about their mental health status and relevant treatments and hospitalizations. Exclusion criteria for participation in the experiment were past or actual presence of mental or neurological disorders (mental health problems, psychiatric treatments and hospitalizations, psychotherapies, neurological problems and treatments, and current use of psychotropic medication). For study inclusion, participants had to be between 18 and 35 years of age. The interviewers were trained, instructed, and supervised by experienced clinical psychologists. Interviews and experiments were conducted on different days. All participants received a financial compensation after completion of the tasks.

The Beck Depression Inventory (BDI-II, German version [36]) was used to assess participants’ depressive symptomatology and to classify them into two groups: participants with no, or only very few, depressive symptoms, and those with depressive symptoms. Grouping was made using the cut-off proposed by Hautzinger et al. [36]. According to their classification of depression severity, total BDI-II-scores between zero and 8 indicate no depression, whereas total scores > 8 indicate at least a minimal level of depression. The study group with depressive symptoms comprised 44 individuals (31 women and 13 men) and the study group without depressive symptoms included 49 individuals (33 women and 16 men).

### Measures

The *Beck Depression Inventory* is a 21-question multiple-choice self-report scale (BDI-II; German version [36]), which assesses the severity and presence of depressive symptoms such as sad mood, concentration problems, suicidal ideation, irritability, loss of interest as well as somatic symptoms during the preceding two weeks. Respondents are asked to rate each item based on four response choices according to the severity of the symptoms, ranging from the absence of a symptom to an intense level (a value of 0 to 3 is assigned to each answer). Higher scores indicate more severe symptoms. The items represent the symptoms of a depressive episode defined by the *Diagnostic and Statistical Manual of Mental Disorders* fourth edition (DSM-IV [37]). In our sample, Cronbach’s alpha was 0.76 for the BDI-II.

The *Differential Emotions Scale* (DES [38]; German version [39]) was applied in its trait form to assess the frequency of experience of basic emotions in everyday life. The DES comprises 30 adjectives (items) and 10 emotion scales (happiness, interest, surprise, and seven negative emotions (i.e., sadness, fear, disgust, contempt, anger, shame, and guilt)). The frequency of emotion experience is assessed on a 4-point scale (0 to 3). The vocabulary of the DES items was derived from an analysis of verbal labels of facial expressions. The DES has been subjected to factor-analysis studies showing that the emotion factors are stable [40, 41]. In the present study, we focused our analysis on the scales assessing happiness, sadness, fear, and disgust, i.e., those emotion qualities, which were shown in the facial expressions during the eye-tracking task. In our sample, Cronbach’s alpha coefficients were acceptable to satisfactory for group comparisons across the scales (happiness: 0.78, sadness: 0.75, fear: 0.65, disgust: 0.64).

The *Multiple-choice vocabulary intelligence test* (Mehrfachwahl-Wortschatz-Intelligenztest, MWT-B [42]) is a performance test, which measures aspects of general intelligence, specifically crystallized, verbal intelligence. The MWT-B consists of 37 items. Each item includes one real word and four pronounceable pseudo-words. It is the subject’s task to identify the real word. The number of correct answers can be transformed into an IQ-score using normative data.

### Free viewing task: face stimuli and procedure

**Fig. 1:**
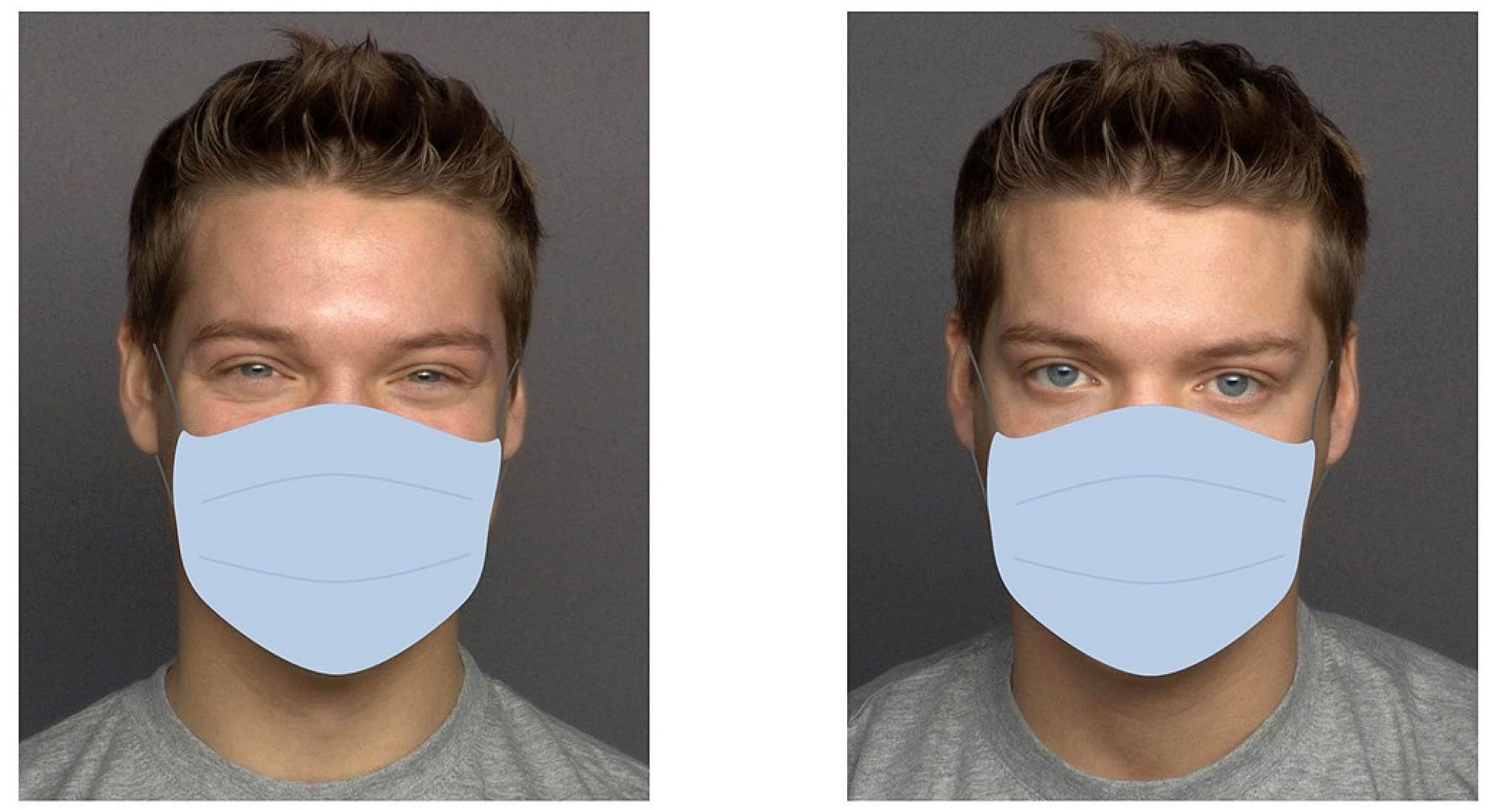
Example of a face pair with face masks administered in the free viewing experiment (happy expression (left) and neutral expression (right)). The original images were taken from the MPI FACES dataset (Ebner et al., 2010). The depicted faces show model 066

In the free viewing task, one-hundred pairs of faces were shown (side by side). An emotional (happy, sad, fearful, or disgusted) or neutral facial expression was presented along with the neutral facial expression of the same model. Facial stimuli comprised one hundred frontal photographs of twenty models (ten women), taken from the MPI FACES dataset [43]. Each model showed five different facial expressions (happy, sad, fearful, disgusted, and neutral). Ten models (five women) were presented without a mask. The photos of ten other models (five women) were digitally edited by superimposing a mask on the original MPI images (see Fig. 1 for an example of a face pair with a face mask). The mask resembled a light blue surgical face mask. It was adapted to match the width and length of the respective face so that it covered the face from the upper nose downwards. Photographs were displayed on a white background. The display size of each face on the screen was 12.4 cm wide x 15.5 cm high. Permission to use and adapt photographs were obtained from the Max Planck Institute for Human Development Berlin.

The free viewing task consisted of 100 trials divided into two blocks. In the first block, face pairs with a face mask were shown (50 trials consisting of 10 happy-neutral faces, 10 sad-neutral faces, 10 fearful-neutral, 10 disgusted-neutral, and 10 neutral-neutral faces). In the second block, faces without a face mask were displayed (50 trials consisting of 10 happy-neutral faces, 10 sad-neutral faces, 10 fearful-neutral, 10 disgusted-neutral, and 10 neutral-neutral faces). In each block, trials were shown in an individual random sequence. The location of stimuli (left-right) was randomized over trials. Camera adjustments were made to best capture participants’ eyes and a nine-point grid was used for calibration, followed by a separate validation using the IViewX software. The calibration was repeated if visual deviation was above 0.7° on the X or Y axis. As mentioned above, two individuals had to be excluded from the study because they did not meet the calibration criteria. Between blocks, participants had a short break. Calibration was done twice, before the start of each block. Each trial of the task started with a fixation cross (gray cross against a white background), shown until a fixation of 1000 ms. Subsequently, a face pair was presented for 5 s. During the experiment, study participants were seated in a chair at approximately 70 cm in front of the screen. The experiment was conducted in a room shielded from sun light. Ceiling lighting produced stable illuminance conditions. All participants were instructed to minimize head and body movements during the experiment. Participants were told that they would see photographs of faces and should view them naturally.

### Eye-tracking: apparatus and eye movement parameters

Eye movements were recorded using an IView X RED250 remote eye-tracker developed by SensoMotoric Instrumens (SMI), which represents an infrared video-based eye-tracking system sampling eye movements every 4 ms (250 Hz) with a high gaze position accuracy (0.4°). A fixation was defined as a stable gaze location within a 1° radius of visual angle with a minimum duration of 100 ms. The SMI RED250 tracker system is able to compensate changes in head position, so that no head resting device is necessary. For data acquisition and stimulus presentation, a SMI-customized Dell laptop (IView X laptop) was used.

**Fig. 2:**
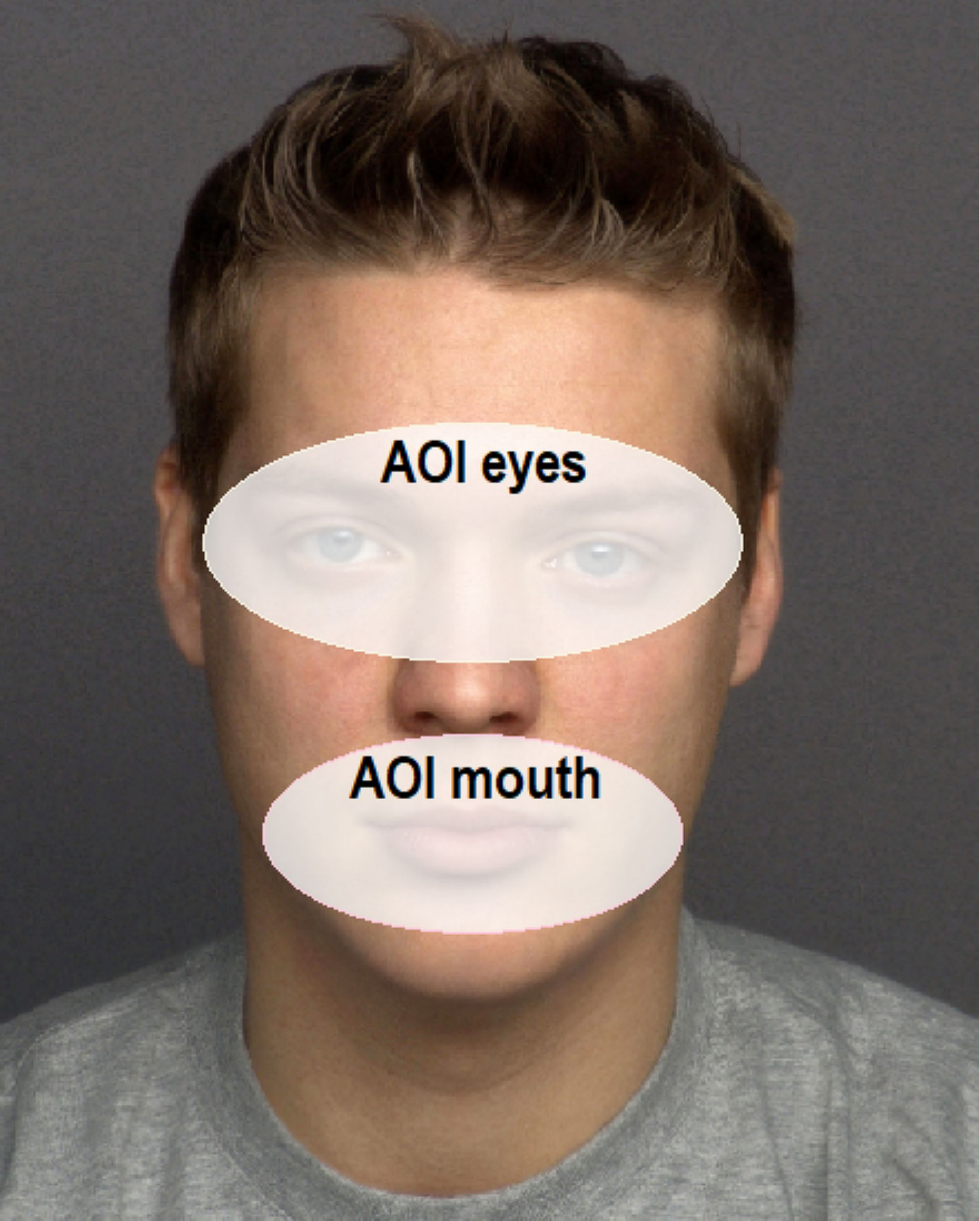
Examples of areas of interest (eyes and mouth) for illustration purposes on a face without face mask. Study participants never actually saw these. The depicted face shows model 066

The SMI software BeGaze 3.0 was employed to define areas of interest (AOI) in each trial; corresponding to the eye and mouth region on each face. Oval-shaped AOIs were created around the eye and mouth region of the faces with the same size and location for each model (see for an example Fig. 2). In addition, an area of interest was created, which comprised the rest of the face (without the AOIs eyes and mouth) consisting of forehead, lower nose, cheeks, and chin. We were primarily interested in assessing the overall time that subjects looked at each AOI. Therefore, we used *dwell time* as an indicator for sustained attention. Dwell time represents the sum of durations from all fixations and saccades that hit the AOI. Moreover, as an index of early or initial attention allocation we used *first fixation duration*. First fixation duration was calculated by averaging the duration of the first fixation on an AOI for each experimental condition.

### Procedure

The experimental session was conducted at the Department of Psychosomatic Medicine and Psychotherapy at the University of Leipzig Medical Center. Study participants were tested individually in a silent room. Due to the COVID-19 pandemic, participants as well as the experimenter wore a face mask throughout the experiment. At the start of the session, study participants filled out the sociodemographic questionnaire and performed the vision test. Next, they had to complete the free viewing eye-tracking experiment. Finally, the psychological tests were administered in the following fixed order: BDI-II, MWT-B, and DES.

### Statistical analyses

Two-sample *t*-tests and Chi^2^-tests were applied to identify differences between study groups in socio-demographic and psychological characteristics. Dwell time data of the eye-tracking task were analyzed in the following way. First, dwell times on the eye region were analyzed by means of a 2 × 2 × 5 mixed ANOVA with study group (individuals with and without depressive symptoms) as between-subjects factor and masking (faces with and without mask) and type of face pair (happiness-neutral, sadness-neutral, fear-neutral, disgust-neutral, and neutral-neutral) as within-subjects factors. Greenhouse-Geisser correction [44] was used to adjust the degrees of freedom of the *F*-ratios when the assumption of sphericity was violated. Follow-up tests were conducted to evaluate pairwise differences (pairwise comparisons). First fixation durations on the eye region were also analyzed by means of a 2 × 2 × 5 mixed ANOVA with study group as between-subjects factor and masking and type of face pair as within-subjects factors. Second, dwell times on the eye and mouth region of faces without face mask were analyzed by means of a 2 × 5 mixed ANOVA with study group as between-subjects factor and type of face pair as within-subjects factor. Third, paired *t*-tests were performed for faces without masks to compare dwell times on the eyes of emotional faces with those on the eyes of paired neutral faces. Fourth, dwell times on the eye region of emotional faces were analyzed using a 2 × 4 mixed ANOVA with study group as between-subjects factor and type of emotion as within-subjects factor.

As the majority of study participants did not look at the mouth AOI in case of faces wearing a face mask we abstained from analyzing these dwell time data. The proportion of study participants who had a dwell time of 0 ms on the mouth AOI in case of faces with face masks was 67% for happy faces, 68% for sad faces, 47% for fearful faces, 52% for disgust faces, and 64% for neutral faces.

Product-moment correlation analyses were performed to explore the relationships between trait emotions (DES scales) and dwell times on eye and mouth regions. Results were considered significant at *p* < 0.05, two-tailed. All calculations were administered using SPSS 29.0 (IBM Corp., Armonk, NY, USA).

## Results

### Sociodemographic and psychological variables

Descriptive statistics for sociodemographic and psychological characteristics as a function of study group are shown in Table 1. Study groups did not differ in age, gender distribution, school education, and intelligence (see Table 1 for details). However, individuals without depressive symptoms had lower BDI-II scores, and reported more trait happiness, and less trait sadness, trait fear, and trait disgust compared to individuals with depressive symptoms (see Table 1).

**Table 1:** Demographic and psychological test data of individuals with and without depressive symptoms (means and SD (in brackets) or frequency values)

Variable	With depressive symptoms (*N* = 44) Mean (SD)	Without depressive symptoms (*N* = 49) Mean (SD)	t / χ^2^	*p*
Age	24.07 (3.86)	24.59 (4.68)	-0.58	0.56
Gender (female/male)	31/ 13	33/ 16	0.10	0.82
School education (years)	12.18 (1.15)	12.20 (0.84)	-0.11	0.91
Intelligence (IQ; MWT-B)	109.64 (11.67)	110.39 (11.62)	-0.31	0.76
BDI-II	13.02 (3.93)	5.04 (2.35)	12.03	< 0.001*
DES Happiness	5.23 (1.60)	6.08 (1.67)	-2.51	0.01*
DES Sadness	2.52 (1.64)	1.31 (1.29)	4.00	< 0.001*
DES Fear	1.61 (1.63)	0.69 (0.89)	3.42	< 0.001*
DES Disgust	0.91 (1.29)	0.37 (0.67)	2.58	0.01*

* Significant differences between groups according to independent samples *t-*tests or χ^2^ tests

MWT-B: Multiple-choice vocabulary test version B; BDI-II: Beck Depression Inventory; DES: Differential Emotions Scale

### Dwell time data

Dwell times as a function of type of face pair, masking, and AOI are presented in Table 2. Trait happiness, trait sadness, trait fear, and trait disgust showed no correlations with any of the dwell time scores (see Supplementary Table S1 for details).

**Table 2:** Dwell times (in ms) on the eye region for happy-neutral, sad-neutral, fearful-neutral, disgust-neutral, and neutral-neutral face pairs with and without face masks and dwell times on the mouth region for face pairs without face masks. Participants with and without depressive symptoms are compared (means with SD (in brackets))

AOI Condition	With depressive symptoms (*N* = 44)	Without depressive symptoms (*N* = 49)
**Eyes in faces without face mask**		
Happy-neutral	1213 (403)	1385 (373)
Sad-neutral	1378 (393)	1533 (377)
Fearful-neutral	1235 (400)	1358 (377)
Disgust-neutral	1286 (405)	1469 (357)
Neutral-neutral	1254 (337)	1382 (363)
**Eyes in faces with face mask**		
Happy-neutral	1881 (237)	1880 (344)
Sad-neutral	1888 (224)	1852 (364)
Fearful-neutral	1772 (257)	1765 (353)
Disgust-neutral	1867 (236)	1839 (369)
Neutral-neutral	1640 (317)	1759 (365)
**Mouth in faces without face mask**		
Happy-neutral	511 (230)	410 (215)
Sad-neutral	311 (177)	236 (187)
Fearful-neutral	351 (168)	274 (181)
Disgust-neutral	357 (212)	279 (196)
Neutral-neutral	300 (184)	253 (165)

A 2 × 2 × 5 mixed ANOVA based on dwell times on the eye region with the factors group, masking, and type of face pair revealed a main effect of masking, *F* (1, 91) = 118.07, *p* < 0.001, ηp² = 0.56. Dwell times on the eyes of faces with face masks were substantially longer than those on the eyes of faces without masks. There was also a significant effect of type of face pair, *F* (3.21, 292.05) = 43.22, *p* < 0.001, ηp² = 0.32, a significant interaction masking x type of face pair, *F* (3.59, 326.88) = 19.17, *p* < 0.001; ηp² = 0.17, and a significant interaction between group, masking, and type of face pair, *F* (3.59, 326.88) = 5.38, *p* < 0.001, ηp² = 0.06. No other effects were significant. To further analyze the three-way interaction, separate two-factor ANOVAs (group x type of face pair) were conducted on dwell times on the eyes for both masking conditions separately. A 2 × 5 ANOVA based on dwell times on the eye region of faces without a mask yielded main effects of type of face pair, *F* (4, 364) = 27.23, *p* < 0.001, ηp² = 0.23, and group, *F* (1, 91) = 4.12, *p* < 0.05, ηp² = 0.043. No further effects were significant. Post-hoc comparisons indicated that individuals without depressive symptoms dwelled longer on the eye region of faces without mask (1426 ms (SD = 355)) compared to individuals with depressive symptoms (1273 ms (SD = 370)). Moreover, dwell times on the eyes of faces without mask in sad-neutral pairs were higher than those on happy-neutral, fearful-neutral, disgust-neutral, and neutral-neutral pairs (*p*s < 0.001). In addition, dwell times on the eyes of faces without mask in disgust-neutral pairs were higher than those on happy-neutral, fearful-neutral, and neutral-neutral pairs (*p*s < 0.01). A 2 × 5 ANOVA based on dwell times on the eye region of faces with face masks showed a main effect of type of face pair, *F* (3.05, 277.22) = 37.30, *p* < 0.001, ηp² = 0.29, and an interaction between group and type of face pair, *F* (3.05, 277.22) = 6.11, *p* < 0.001, ηp² = 0.063. No further effect was significant. Post-hoc comparisons indicated that individuals without depressive symptoms tended to dwell longer on the eye region of faces with masks in neutral-neutral pairs (1759 ms (SD = 365)) compared to individuals with depressive symptoms (1640 ms (SD = 317)), *p* < 0.098. For the other face pair conditions, dwell times on the eyes were very similar between study groups. Dwell times on the eyes of faces with masks in happy-neutral pairs were higher than those on fearful-neutral, disgust-neutral, and neutral-neutral pairs (*p*s < 0.05). In addition, dwell times on the eyes of faces with masks in sad-neutral and disgust-neutral pairs were higher than those on fearful-neutral, and neutral-neutral pairs (*p*s < 0.001). Finally, dwell times on the eyes of faces with masks in fearful-neutral pairs were higher than those in neutral-neutral pairs (*p*s < 0.01).

**Fig. 3:**
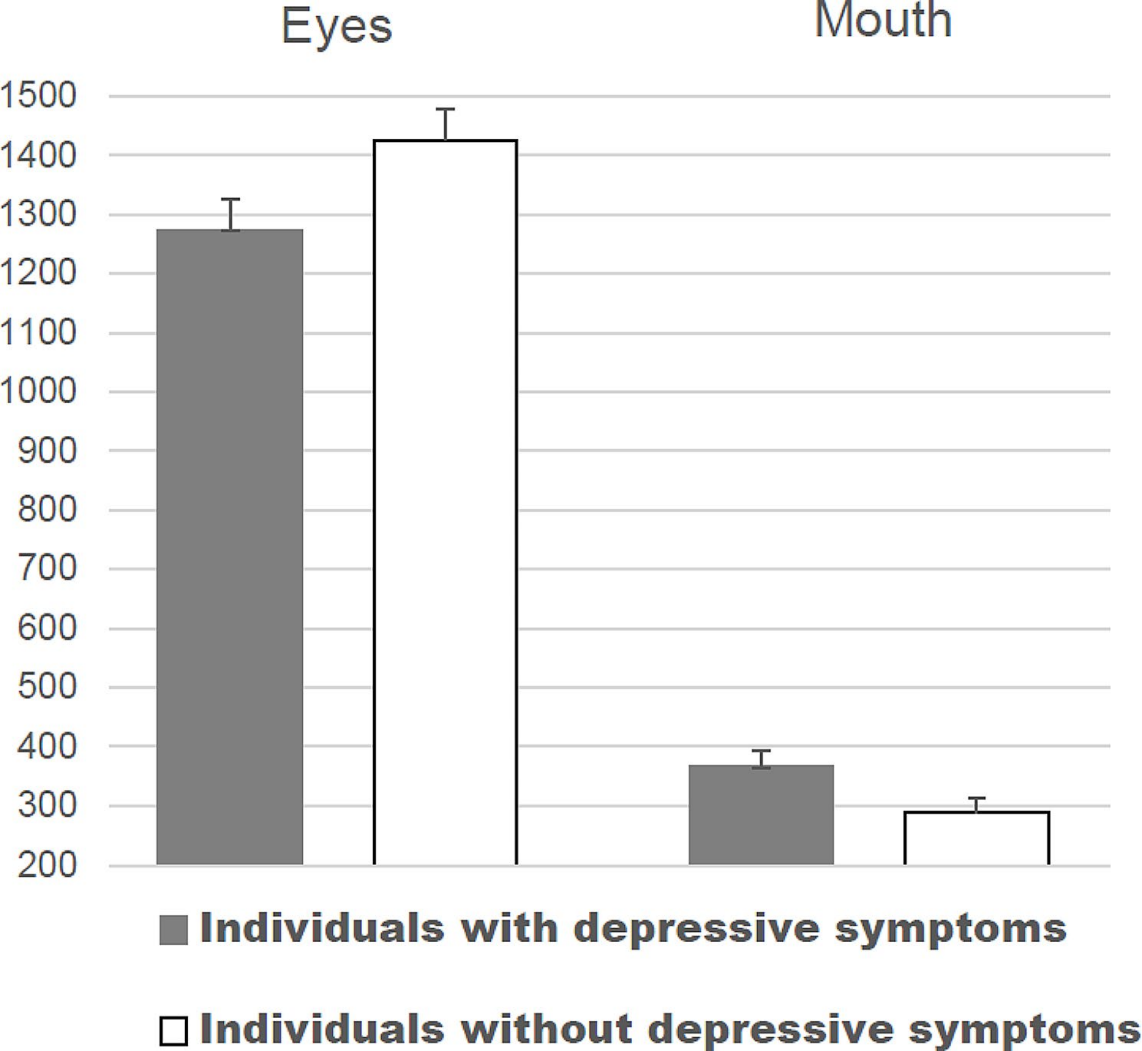
Dwell times (in ms) of study groups on the eye region and the mouth for face pairs without face masks (means with standard error)

A second series of ANOVAs were performed to examine group differences in dwell time on the eyes and the mouth in faces without masks. A 2 × 2 × 5 mixed ANOVA based on dwell times with the factors group, AOI, and type of face pair revealed significant main effects of AOI, *F* (1, 91) = 390.41, *p* < 0.001, ηp² = 0.81, and type of face pair, *F* (3.42, 311.55) = 36.77, *p* < 0.001, ηp² = 0.29. Moreover, the interactions group x AOI, *F* (1, 91) = 4.87, *p* < 0.05, ηp² = 0.05, and AOI x type of face pair were significant, *F* (3.57, 324.49) = 43.50, *p* < 0.001, ηp² = 0.32. No further effects were significant. The results of post-hoc comparisons indicated that individuals without depressive symptoms dwelled longer on the eye region of faces without mask compared to individuals with depressive symptoms (*p* < 0.05) and that individuals with depressive symptoms dwelled longer on the mouth region (366 ms (SD = 176)) compared to individuals without depressive symptoms (290 ms (SD = 175)), *p* < 0.05 (see Fig. 3). An additional analysis based on overall dwell times on the rest of the face (without the AOIs eyes and mouth) consisting of forehead, lower nose, cheeks, and chin yielded no differences between individuals with depressive symptoms (1188ms (SD = 304)) and individuals without depressive symptoms (1246ms (SD = 284)), *t* (91) = 0.95, *p* = 0.34.

The results of paired *t*-tests indicate that, for faces with no mask, dwell times on the eyes of emotional faces were significantly longer than those on the eyes of the paired neutral faces (happy (1587 ms (SD = 585)) vs. neutral (1021 ms (SD = 393)), sad (1763 ms (SD = 595)) vs. neutral (1155 ms (SD = 489)), fearful (1561 ms (SD = 613)) vs. neutral (1039 ms (SD = 434)) and disgusted (1602 ms (SD = 600)) vs. neutral (1162 ms (SD = 510))), *p*s < 0.001.

Dwell times on the eye region for happy, sad, fearful, and disgust faces without face masks are shown as a function of study group in Table 3. A 2 × 4 mixed ANOVA with study group as between-subjects factor and type of emotion as within-subjects factor revealed a significant main effect of emotion, *F* (2.65, 240.88) = 13.48, *p* < 0.001, ηp² = 0.13. No other effect was observed. Dwell times on the eyes of sad faces were higher than those on the eyes of happy, fearful, and disgusted faces, *p*s < 0.001.

**Table 3:** Dwell times (in ms) on the eye region for happy, sad, fearful, and disgust faces without face masks. Participants with and without depressive symptoms are compared (means with SD (in brackets))

Facial expressions	With depressive symptoms (*N* = 44)	Without depressive symptoms (*N* = 49)
Happy	1444 (550)	1715 (591)
Sad	1670 (559)	1846 (619)
Fearful	1507 (621)	1609 (608)
Disgust	1526 (584)	1671 (612)

### First fixation duration data

Durations of first fixation on the eyes as a function of type of face pair, and masking are shown in Table 4. A 2 × 2 × 5 mixed ANOVA based on first fixation durations on the eye region with the factors group, masking, and type of face pair revealed only a main effect of type of face pair, *F* (4, 364) = 3.42, *p* < 0.01, ηp² = 0.04. According to post-hoc comparisons first fixation durations on the eyes in disgust-neutral pairs (346ms) were longer than those in happy-neutral pairs (326ms) (*p* < 0.05). No other significant differences between type of face pairs were observed. Importantly, no other ANOVA effects were significant.

**Table 4:** First fixation durations (in ms) on the eye region for happy-neutral, sad-neutral, fearful-neutral, disgust-neutral, and neutral-neutral face pairs with and without face masks. Participants with and without depressive symptoms are compared (means with SD (in brackets))

AOI Condition	With depressive symptoms (*N* = 44)	Without depressive symptoms (*N* = 49)
**Eyes in faces without face mask**		
Happy-neutral	314 (92)	346 (122)
Sad-neutral	352 (126)	338 (148)
Fearful-neutral	320 (93)	348 (134)
Disgust-neutral	343 (130)	363 (139)
Neutral-neutral	333 (114)	338 (114)
**Eyes in faces with face mask**		
Happy-neutral	324 (92)	322 (93)
Sad-neutral	344 (98)	339 (127)
Fearful-neutral	323 (93)	325 (118)
Disgust-neutral	333 (106)	346 (137)
Neutral-neutral	340 (92)	317 (97)

## Discussion

In the present study, we investigated whether depressive symptoms in healthy individuals are related to reduced visual attention to other persons’ direct gaze. Visual attention was primarily defined as the time study participants’ gaze dwelled on others’ eyes. In our free viewing experiment, pairs of faces with a direct gaze and emotional or neutral expressions were shown side by side. In the experiment, one half of the face pairs was displayed without face masks, whereas the other half wore face masks, which resembled surgical masks covering mouth and parts of the nose. The present results partially confirm our first hypothesis: in case of unmasked facial expressions (where all facial features are clearly visible), individuals with depressive symptoms look shorter at other persons’ eyes compared to individuals without depressive symptoms across all expression conditions. Instead, individuals with depressive symptoms dwelled longer on the mouth region. No group differences were found for dwell time on the rest of the face, an AOI comprising forehead, lower nose, cheeks, and chin. This pattern of results suggests that individuals with depressive symptoms do not manifest generally less visual attention to others’ faces but that they hold their gaze less on others’ eyes. This means that in case of unmasked faces with gaze directed at the beholder presence of depressive symptoms might diminish the attention being paid to the others’ eyes. It is interesting to note that trait sadness (but also trait happiness, trait fear and trait disgust) were not related to dwell time on the eyes in our study. Thus, it appears that current depressive symptoms could be more relevant in the management of visual attention to the eyes than the general disposition to experience sadness in everyday life.

For faces wearing face masks, our findings do not support the hypothesis that individuals with depressive symptoms look shorter at other persons’ eyes compared to individuals without symptoms. No significant group differences concerning dwell times on the eye region were found for the masked face pairs. In this context, it is important to consider that, as hypothesized, when our participants looked at faces with face masks their dwell time on the eyes was substantially increased compared to that when they looked at unmasked faces. These findings are in line with those of Rabadan et al. [35] who reported that people move their gaze preferentially to the eyes of faces in which the lower part is hidden by a scarf or mask. In our study, face masks did not attract participants’ attention. In considerably more than 50% of all trials with masked faces participants did not look at the mouth region (AOI) at all. This is not surprising since the face masks shown were very similar among each other and had a poorly contoured surface. Thus, in a setting where the lower part of the face is covered, and participants’ gaze is strongly directed towards the upper part of the face the effect of current depressive symptoms on the orientation and maintenance of gaze could be attenuated.

According to our data, participants dwelled longer on the eyes of unmasked emotional faces than on the eyes of the paired neutral faces. This means that when looking at pairs of unmasked faces consisting of an emotional and a neutral one participants’ attention was preferentially allocated to the eyes of the emotional faces regardless of emotional quality. These results are consistent with previous research showing that in general emotional facial expression accelerates engagement and delays disengagement of attentional resources due to its high communicative significance [45]. From an evolutionary perspective, it makes sense for humans to prioritize emotional facial expressions during perception because it allows them to respond quickly to potential challenges in their environment [46]. In addition, we found that participants dwelled longer on the eyes of sad faces than on the eyes of happy, fearful, and disgusted faces. Eye-tracking research has shown that during processing of emotional facial expressions gaze is primarily directed towards two diagnostic facial features: the eyes and the mouth [3, 47]. The diagnostic importance of these features, however, varies as a function of the specific emotion expressed. For sad facial expressions, it was observed that the eyes are more often fixated in comparison with other emotional expressions [47]. Attention to the eyes decreases for happy and disgusted facial expressions for which lower parts of the face are diagnostically more important [48, 49]. For facial expressions of fear, both the eye and the mouth region appear to attract attention [50, 51]. Thus, our results are in accord with previous findings suggesting that the eyes receive attention for longer when viewing sad facial expressions compared to other facial emotions. However, we found no evidence for emotional facial expressions that individuals with depressive symptoms look shorter at the eyes compared to individuals without symptoms. It means that depressive symptoms seem not to be associated with less eye contact when people watch faces expressing emotions.

To summarize, the findings of our study suggest that depressiveness in healthy individuals goes along with less visual attention to other persons’ eyes but not with less attention to others’ faces. However, when factors come into play that generally amplify the attention directed to the eyes such as face masks or emotions expressed by the model then the relationship between depressiveness and reduced visual attention to the eyes is no longer detectable. Depressiveness might have a modulating effect on duration of attention paid to the eyes in case faces are completely visible. The present results agree with those of previous mood induction studies with healthy individuals, which found that sad individuals had less eye contact during conversations than individuals in a neutral affective state [27] or were less likely to attend to the eyes during face perception than happy persons [28]. Prior clinical studies yielded analogous results: depressed patients were found to make less eye contact during interviews [22, 23]. In this context, it has been argued that avoidance of eye contact during social interaction could contribute to social functioning impairments in depression [52]. Keeping the gaze away from other persons’ eyes could be part of social withdrawal tendencies in depression [53] since eye contact not only represents a key signal in the initiation of conversations [5] but also communicates social interest and closeness [11, 12]. It is known that eye contact enhances self-referential processing [17]. Since depressive thinking is characterized by persistent negative self-evaluations [54] it is possible that depressed individuals are motivated to reduce eye contact to avoid an intensification of burdensome thoughts related to the self. According to the social competition hypothesis [55] depression represents a state of submission and helplessness (see also [56]). Depressive symptoms in healthy individuals have been found to be linked to experiences of low self-esteem and low social rank [57, 58]. Against this background the association of depressive symptoms with reduced visual attention to other persons’ eyes observed in our study might also be interpreted as reflecting depressive state related tendencies of submissive gaze aversion [59].

The present free viewing eye-tracking study contributes to the literature on depression and social perception indicating that presence of depressive symptoms may decrease attention to other persons’ eyes in healthy individuals. Our investigation was inspired by previous research findings of reduced eye contact in clinically depressed patients, which were based on video recordings of conversations or involved assessors of eye contact registration. The present eye-tracking study examined processes of attention allocation to the eyes in static photos of faces so that we cannot claim to have assessed eye contact (with a real person). Our investigation using a free viewing task complements and expands previous eye-tracking research on depressive symptomatology and gaze behavior during facial emotion recognition [29, 30]. In the latter studies, depressive symptoms were found to be related to less attention to various central facial features, in particular middle facial areas (eyes and cheekbones) when identifying facial emotions. Our free viewing results suggest instead that the reduction of visual attention associated with depressive symptoms is specific to the eyes and observed only for late or sustained attention but not for early processes of attention allocation (as assessed by first fixation duration).

Further eye-tracking studies should follow to test the robustness of our results and to extend them to clinically depressed populations. Moreover, it appears methodologically useful to compare gaze behavior towards faces with direct and averted gaze in depression. Seeing other persons’ averted gaze signals their attention to be directed away from oneself [60] so that no direct eye contact can be established which may encourage depressed individuals to explore the others’ eye region more intensely. This explorative eye-tracking study revealed a difference in visual attention to others’ eyes for unmasked faces between individuals with and without depressive symptoms, which according to Cohen’s conventions is a small to medium effect (*d* = 0.422). A post hoc analysis of statistical power was conducted using the program G*Power (version 3.1.9.2 [61]. ; differences between two independent means - two groups). To detect an effect of *d* = 0.422 with an alpha value of 0.05, one-tailed, and sample sizes of N_1_ = 44 and N_2_ = 49, the achieved power is 0.65. It is recommended that future studies on the current topic should increase number of participants per study group to achieve a more satisfactory statistical power with the observed effect size. An a priori power analysis computing required sample size to achieve a power of 0.80 (with an alpha value of 0.05) yielded a sample size of 71 per group.

Finally, a number of limitations must be acknowledged in the current work. The generalizability of the results is restricted by the fact that study participants were well-educated young individuals. Thus, additional studies are required to replicate the findings in populations other than university students. In our free viewing task, static photographs of face pairs were administered. In future research, videos of emotional facial expressions should be used to investigate whether dynamic facial stimuli yield similar results. Dynamic emotional facial expressions have higher ecological validity than still pictures in studies on eye contact. Even more informative could be research based on mobile eye-tracking glasses, which enables to examine gaze behavior during natural social interactions directly. However, in this context, it should be noted that there exist associations between gaze patterns during face perception in screen-based laboratory experiments and those observed in real-world settings [62]. Individual variations in face gaze behavior were also found to be quite robust across screen-based and live interview scenarios [63]. A further methodological limitation of our study represents the fixed order in which masked and unmasked faces were shown. In the first block, face pairs with a face mask were displayed, whereas in the second block, faces without a face mask were shown. The order of experimental tasks could have an effect on participants’ free viewing behavior - in particular in the unmasked face condition. Future eye-tracking research showing masked and unmasked faces should systematically vary the sequence of stimulus presentation and examine order effects.

### Electronic supplementary material

Below is the link to the electronic supplementary material.

Supplementary Material 1

## Data Availability

The datasets used and/or analyzed during the current study are available from the corresponding author on reasonable request.

## Abbreviations

AOI: Area of interest
BDI-II: Beck Depression Inventory
DES: Differential Emotions Scale
IQ: intelligence quotient
MWT-B: Multiple-choice vocabulary test version B
