# Supplementary material for: Depressive symptoms and visual attention to others’ eyes in healthy individuals
Source: BMC Psychiatry. 2024 Mar 6;24:184. doi: 10.1186/s12888-024-05633-2 (PMC10916197; doi:10.1186/s12888-024-05633-2)
Supplement: Supplementary file 1 — Supplementary Material 1 [file 12888_2024_5633_MOESM1_ESM.docx]

**Supplementary Table S1**

Product-moment correlations between trait emotions as assessed by the Differential Emotions Scale and dwell times in the different AOI conditions (eye region for face pairs with and without face masks and mouth region for face pairs without face masks) (*N* = 93).

| **AOI Condition** | Happiness | Sadness | Fear | Disgust |
| --- | --- | --- | --- | --- |
| **Eyes in faces without face mask** |  |  |  |  |
| Happy-neutral | .05 | -.01 | .03 | -.14 |
| Sad-neutral | .01 | -.02 | .07 | -.04 |
| Fearful-neutral | .00 | -.04 | .05 | -.05 |
| Disgust-neutral | .05 | -.03 | .14 | -.04 |
| Neutral-neutral | .08 | -.10 | -.02 | -.18 |
| **Eyes in faces with face mask** |  |  |  |  |
| Happy-neutral | -.06 | -.03 | .06 | .04 |
| Sad-neutral | -.11 | .01 | .06 | .12 |
| Fearful-neutral | -.18 | .05 | .13 | .07 |
| Disgust-neutral | -.15 | .02 | .12 | .17 |
| Neutral-neutral | -.10 | -.08 | .01 | -.03 |
| **Mouth in faces without face mask** |  |  |  |  |
| Happy-neutral | .03 | .02 | .00 | .06 |
| Sad-neutral | .07 | .09 | -.02 | -.04 |
| Fearful-neutral | .03 | .14 | -.02 | .04 |
| Disgust-neutral | .02 | .08 | -.09 | .00 |
| Neutral-neutral | .04 | .05 | -.01 | -.05 |
